# Microfluidic Generated EGF-Gradients Induce Chemokinesis of Transplantable Retinal Progenitor Cells via the JAK/STAT and PI3Kinase Signaling Pathways

**DOI:** 10.1371/journal.pone.0083906

**Published:** 2013-12-23

**Authors:** Uchenna J. Unachukwu, Moira Sauane, Maribel Vazquez, Stephen Redenti

**Affiliations:** 1 Biochemistry Doctoral Program, The Graduate School and University Center, City University of New York, New York, New York, United States of America; 2 Department of Biological Sciences, Herbert Lehman College, City University of New York, Bronx, New York, United States of America; 3 Department of Biomedical Engineering, City College of New York, City University of New York, New York, New York, United States of America; 4 Biochemistry Doctoral Program, The Graduate School and University Center, City University of New York, Department of Biological Sciences, Herbert Lehman College, City University of New York, Bronx, New York, United States of America; University of California, Irvine, United States of America

## Abstract

A growing number of studies are evaluating retinal progenitor cell (RPC) transplantation as an approach to repair retinal degeneration and restore visual function. To advance cell-replacement strategies for a practical retinal therapy, it is important to define the molecular and biochemical mechanisms guiding RPC motility. We have analyzed RPC expression of the epidermal growth factor receptor (EGFR) and evaluated whether exposure to epidermal growth factor (EGF) can coordinate motogenic activity *in vitro*. Using Boyden chamber analysis as an initial high-throughput screen, we determined that RPC motility was optimally stimulated by EGF concentrations in the range of 20-400ng/ml, with decreased stimulation at higher concentrations, suggesting concentration-dependence of EGF-induced motility. Using bioinformatics analysis of the EGF ligand in a retina-specific gene network pathway, we predicted a chemotactic function for EGF involving the MAPK and JAK-STAT intracellular signaling pathways. Based on targeted inhibition studies, we show that ligand binding, phosphorylation of EGFR and activation of the intracellular STAT3 and PI3kinase signaling pathways are necessary to drive RPC motility. Using engineered microfluidic devices to generate quantifiable steady-state gradients of EGF coupled with live-cell tracking, we analyzed the dynamics of individual RPC motility. Microfluidic analysis, including center of mass and maximum accumulated distance, revealed that EGF induced motility is chemokinetic with optimal activity observed in response to low concentration gradients. Our combined results show that EGFR expressing RPCs exhibit enhanced chemokinetic motility in the presence of low nanomole levels of EGF. These findings may serve to inform further studies evaluating the extent to which EGFR activity, in response to endogenous ligand, drives motility and migration of RPCs in retinal transplantation paradigms.

## Introduction

The loss of retinal tissue due to trauma or disease such as age-related macular degeneration (AMD) remains a tragic and largely untreatable problem. More than 1.6 million Americans have AMD [1]. In cases of significant loss of neural retina there is permanent vision loss with no available restorative treatment. Cell replacement strategies for retinal tissue have been shown to be feasible in animal models using retinal progenitor cells (RPCs) derived from embryonic stem cells (ESCs), neural progenitor cells (NPCs), induced pluripotent stem cells (iPSCs), or early postnatal retina [2,3]. Current retinal transplantation paradigms utilize either bolus injection or delivery of new cells on biodegradable substrates [4-7]. For restoration of retinal architecture and visual function, transplanted RPCs have to migrate from the point of transplantation, commonly the sub-retinal space, through the interphotoreceptor matrix (IPM) to integrate into appropriate lamina [3,8,9]. Unfortunately, the migration of transplanted RPCs in retinal tissue is limited in both healthy/control and diseased retina [5-7,10]. Restricted retinal migration has been shown for RPCs isolated from retina [5,11,12] and photoreceptor progenitors derived from ES and iPSCs *in vitro* prior to transplantation [13]. The molecular and biochemical mechanisms underlying the migration of transplanted RPCs to retinal lamina are poorly understood. Identified barriers to RPC migration and integration include a meshwork of chondroitin sulfate proteoglycans in the IPM, inhibitory extracellular matrix (ECM) molecules and obstructive microglial accumulation [14]. Progenitors can dissolve the ECM by releasing matrix metalloproteinases and experimental disruption of structural barriers can modestly enhance RPC integration [4,15,16]. In this study, we begin the process of delineating RPC surface receptors and endogenous extracellular factors capable of enhancing the directed motility of transplanted RPCs. The characterization of molecular mechanisms and biochemical compounds capable of guiding successful migration of RPCs in the retina is essential for increasing the efficacy of future transplantation strategies.

In adult human and mouse retina, constitutive expression of the EGFR has been observed in ganglion, amacrine and horizontal cells, and synaptic regions of photoreceptors [17-19]. Increased EGFR expression has been described in human retina during proliferative diabetic retinopathy [20]. Adult human and mouse retinal ganglion cells have been shown to transcribe EGF mRNA [18,19]. In addition, in damaged retina, Muller glia up-regulate synthesis of EGF to levels similar to those driving histogenesis during development [17]. 

The activation of EGFR has been associated with the proliferation and motility of RPCs, a range of neural progenitors [17,21-24] and retinal pigment epithelial cells [25]. RPCs transplanted into adult retina are localized to a region of interphotoreceptor matrix, photoreceptor outer segments and Muller glia end-feet [26]. As ganglion and Muller glia have been shown to synthesize EGF, it is plausible that a chemotactic gradient of EGF is established, influencing migration of EGFR-expressing RPCs. Growth factor gradients have previously been shown to stimulate proliferation and motility of neural progenitors [27], connective tissue-derived cells [28], and cancer cells [29,30] and are utilized in many related biological applications [31]. To determine the role of EGF gradients on RPC motility, steady-state nanomolar level gradients of EGF were generated in this study using a bioengineered microfluidic system [32].

For cell motility to be elicited, EGF binds to its receptor on the plasma membrane, induces dimerization of EGFR which activates its tyrosine kinase, auto-phosphorylation and/or internalization of receptor-ligand complexes [33]. Mediators such as phospholipase C-γ1 (PLC-γ1), focal adhesion kinase (FAK) and Rho-GTPases signal canonical downstream pathways including the PI3Kinase [24] and MAPK signaling pathways [34] responsible for disruption of focal adhesions and stimulation of cytoskeletal reorganization to facilitate cell motility [35-37]. 

In this work, we used bioinformatics to map intracellular EGF signaling pathways in RPCs, selectively inhibited resolved pathway molecules, and evaluated their influence on motility. Bioinformatics-modeled gene networks have previously been shown to successfully predict and target molecular interactions *in vitro* and *in vivo* [38,39]. Here, RPC migratory dynamics were analyzed in response to nanomolar EGF concentrations alone and in the presence of pharmacologic modulators of genetic pathways [40]. Stimulation and inhibition parameters were initially screened using high-throughput Boyden assays. In the presence of optimized exposure time and concentration parameters, individual RPC migratory dynamics were analyzed in bioengineered microfluidic devices with defined quantifiable EGF gradients. Our results show that low nanomole EGF concentration ranges stimulate activity of JAK-STAT and PI3K pathways resulting in increased chemokinesis of RPCs. The results shown in this study further our understanding of molecular and biochemical interactions necessary for RPC motility and may help guide the development of optimized cell-replacement transplantation paradigms.

## Materials and Methods

### Retinal Progenitor Cell Culture

All experiments were approved by and performed in compliance with the City University of New York, Lehman College Animal Care and Use Committee (IACUC). Retinal progenitor cells (RPCs) were isolated from postnatal day (PN) 0-3 mouse retina and maintained in culture as previously described [41]. PN 0-3 Beta actin-GFP^+^ RPCs were cultured in Neurobasal (NB) complete culture medium containing 2% B-27, 1% L-glutamine, 1% Pen Strep, 1% N2 (50X), 2% Nystatin, and 93% NB only (Invitrogen-Gibco, Rockville, MD) and 20ng/ml epidermal growth factor (Promega, Madison, Wisconsin). In preparation for experiments, RPCs were rinsed and cultured without EGF in NB complete culture medium. RPCs were maintained in 5% CO_2_ at 37°C during all experiments. 

### IPA Bioinformatics Analysis

A signaling interactions network specific for the EGF ligand was generated using Ingenuity Pathway Analysis (IPA) (Ingenuity® Systems, www.ingenuity.com, Redwood City, CA) knowledge base. Complex molecular networks orchestrating cellular decision-making are guided by changes in intracellular and extracellular micro-environmental signaling. A major strength of IPA software is its comprehensive regularly updated database of experimentally predicted molecular signaling networks. An increasing number of studies are utilizing IPA database network analysis to predict cell and tissue specific signaling interactions [39,42]. In this study, EGF was entered into the IPA platform as the ligand target. Direct and indirect molecular interactions associated with EGF were generated from the IPA network database. Molecules in the network were further specified for their activity in the mammalian retino-neural system followed by a functional analysis to identify the biological functions most relevant to the resulting molecules in the network. A right-tailed Fisher’s exact test was used to calculate a p-value determining the probability that each biological function assigned to that network was due to chance alone. 

### Cell Proliferation Assay

A cell proliferation assay (Vybrant MTT, Invitrogen, Grand Island, NY) was carried out to examine RPC growth in different EGF concentrations for the same 24hr duration used in cell motility experiments. This assay was carried out to ensure that EGF ligand concentration effects measured were for motility only, distinguishable from cell proliferation. In n=6 wells per condition, approximately 5000 RPCs per well were incubated in NB with 5% CO_2_ at 37°C overnight in duplicate 96-well culture plates and in quadruplicate wells/plates. Media was then aspirated and replaced with 100μl of NB media supplemented with: 20, 40 and 400ng/ml EGF concentrations. Wells were incubated for 24hrs, after which 10μl of 10% MTT reagent was added for 4hrs followed by 100μl of MTT solubilization buffer overnight. Absorbance was measured at 575nm and 650nm using a Synergy Mx plate reader (BioTek Instruments Inc., Winooski, VT), and results plotted against EGF concentrations after background correction. Using serial dilution with NB media, the absorbance of increasing numbers of RPCs ranging from 1000 to 20000 cells/well, were used to create a standard curve correlating cell number to absorbance units. Using a Dunnett statistical test, no significant differences in cell numbers were observed between control and experimental conditions (n=6 wells each; mean ± SD): 0ng = 9431.1± 1163.68 (p=1.0000), 20ng = 9219.5 ± 713.36 (p=0.9611), 40ng = 9388.2 ± 669.46 (p=0.9966) and 400ng= 8779.8 ± 1544.60 (p=0.4956). 

### Immunocytochemistry

For each antibody evaluated, RPCs were seeded onto n=6 laminin-coated (10µg/mL) coverslips (15mm (19/32”), Thermoscientific, Rochester NY) at a density of 9,000 to 13,000 cells/cm^2^ in 6-well culture plates and incubated overnight at 37°C for immunocytochemical staining using a previously described protocol [43]. Briefly, adherent cells were rinsed twice with warm phosphate-buffered saline (PBS) and fixed for 10mins in 1% paraformaldehyde in PBS. After rinsing with wash buffer (0.1% BSA in PBS), cells were blocked and permeabilized for 45 mins by incubation in 0.3% Triton X-100 and 10% normal donkey/rabbit serum (Sigma-Aldrich, St. Louis, MO). Primary antibodies specific to either total epidermal growth factor receptor, which labels both phosphorylated and non-phosphorylated EGFR, (anti-EGFR-total) (1:1000, Sigma-Aldrich, St. Louis, MO) or phosphorylated epidermal growth factor (anti-EGFR-phospho) (1:1000, Abcam, Cambridge, MA) antibodies were used for ICC receptor localization [44,45] Antibodies were diluted in incubation buffer (1% BSA plus 1% normal donkey/rabbit serum plus 0.3% Triton X-100 and 0.01% sodium azide in 1X PBS) and incubated with coverslips overnight at 4°C. Coverslips with adherent cells (n = 6) per antibody were then washed thrice (5 min each) with wash buffer and subsequently incubated with secondary anti-mouse TritC-conjugated antibody (1:10000; R&D systems, Minneapolis, MN) for phospho- and total EGFR receptors, for 1 h at room temperature. After rinsing once for 15 min and thrice for 5 min with wash buffer, cover slips were inverted onto glass slides coated with DAPI-containing anti-fade mounting medium (Invitrogen, Grand Island, NY). DAPI-stained nuclei, GFP^+^ cell body and TritC-conjugated EGF receptor expression were assessed on an inverted fluorescent microscope (Nikon Eclipse Ti, Melville, NY) using 40X and 100X objectives. 

### Western Blot

Ice cold lysis buffer (0.5ml per 5 X 10^6^ cells) containing: 150mM NaCl, 1% Triton X-100, 50 mM Tris, pH 8.0, 1 mM phenylmethylsulfonyl fluoride (PMSF, Sigma-Aldrich, P7626), Halt Protease inhibitor Cocktail (Pierce IL, 78425), and phosphatase inhibitor (Sigma-Aldrich, P5726), was added to RPC cultures that had been incubated for 24hrs with 0, 20 and 40ng/ml EGF concentrations. The cell mixture was agitated for 30mins and then centrifuged at 12,000rpm for 20mins at 4°C. The resulting supernatant was used to determine protein concentration by the Bradford assay and subsequently, 25-40 µg of protein was separated on 8% SDS-PAGE (Clear Page Gel, CBS Scientific, San Diego, CA) and then transferred onto nitrocellulose membranes. The membranes were probed overnight at 4°C with monoclonal anti-EGFR (Sigma-Aldrich, St. Louis, MO) and anti-phospho-EGFR (Abcam, Cambridge, MA), polyclonal anti-ERK1/2 (Santa Cruz Biotech., Dallas, TX), anti-PI3K, anti-β-tubulin, polyclonal anti-phospho-STAT3 (Cell Signaling Technology, Danvers, MA), and polyclonal anti-STAT3 (Calbiochem, Darmstadt, Germany) primary antibodies. Secondary antibodies conjugated to horse-radish peroxidase (HRP) (1:10000, Jackson ImmunoResearch, Westgrove, PA) was bound to the nitrocellulose membranes for 1hr at room temperature and X-ray detection was carried out after applying enhanced chemiluminescence substrate mixture (ECL Plus; Pierce IL) to the membrane. β-tubulin was used to correct for blotting efficiency and normalization. Similar methods used for detecting intracellular signaling activity have previously been described [46].

### Boyden Chamber Migration Assay and Motility-inhibition studies

Initial screening for chemotactic response of RPCs to epidermal growth factor (EGF) was assessed using the Boyden chamber assay as previously described [24,46-49]. Cells were grown in neurobasal (NB) media containing 2% B-27, 1% L-glutamine, 1% Pen Strep, 1% N2 (50X), 2% Nystatin, and 93% NB only. RPCs were then re-suspended in NB media complete supplemented with 10% fetal bovine serum (FBS) and seeded at a density of 1 X 10^5^ cells in 350µl volumes on the upper chamber of non-coated polyethylene terephthalate (PET) membrane filters (8µm pore size, BD Falcon, NJ). These filters insert into tissue culture wells (BD Falcon, NJ) containing 0 (control), 20, 40 and 400ng/ml EGF diluted in 700µl volumes of NB media. Boyden chamber incubation period optimization studies for 6-48hrs were performed using 40ng/ml EGF and pooled data from N=24 chambers were reported as normalized mean ± SEM: 6hrs = 0.193 ± 0.05 p=0.6749, 18hrs = 1.29 ± 0.18 p=0.1067, 24hrs = 1.97 ± 0.30 p=0.0026* and 48hrs = 1.87 ± 0.21 p=0.0026*. To ensure optimal ligand stimulation and cell viability, the 24hr time-point was selected. In the experiments in which inhibitors to EGFR or selected intracellular signaling proteins were used, RPCs were pre-incubated with the inhibitors at 37°C in 5% CO_2_ before loading in the upper chamber as has been previously described [22,24,46]. Inhibitor concentrations and their incubation times with RPCs in Boyden Chambers included the following: (1) monoclonal anti-EGFR (100nM, 2hrs, Sigma-Aldrich Co., St. Louis, MO), (2) STAT3 inhibitor, AG 490 (5μM, 60mins, Calbiochem, Darmstadt, Germany), (3) the cell-permeable, irreversible and selective inhibitor of EGFR tyrosine kinase activity Tyrphostin AG1478 (200nM, 30mins), (4) ERK1/2 inhibitor PD98059 (10μM, 60mins) and (5) PI3K inhibitor Wortmanin (250nM, 60mins), all from Cell Signaling Technology, Danvers, MA. RPCs were then allowed to migrate for 24hr at 37°C and 5% CO_2_. Filters were fixed with 4% paraformaldehyde in PBS for 5 min and stained with DAPI or Hoechst 33342 nuclear stains for 10 min. Cells are scraped from the upper chamber, and the number of stained migrated cells at the bottom surface of the filter were counted at ﬁve ﬁelds per filter for triplicate filters in two independent experiments (n=6) using an inverted fluorescent microscope. Control tissue culture wells contained NB media without any added chemotactic factor (migration study) or without inhibitors (inhibition study). A Dunnett statistical test was used to determine the number of migrated RPCs normalized to cell migration in control filters. Successful inhibition of RPC migration was determined by RPC numbers in test wells that were significantly (p<0.05) less than cell numbers in control chambers containing no inhibitors.

### The Bridged µ-lane Microfluidic System

A micro-fabricated system, bridged μ-lane [32] was utilized to live-image individual RPC chemotactic responses within microenvironments that create precise spatial and temporal profiles of EGF concentration gradients over long distances. The bridged μ-lane device applies buoyancy-driven forces to initiate minute convective velocities that assist molecular diffusion of chemotropic factors. Quantification of ligand mass transport within the microsystem was solved using finite element methods (FEMLab Version 3.4, Comsol Inc., Burlington, MA). Sequential optical monitoring of individual cell movement within predictable microenvironments in the device permitted measurement of mechanistic parameters governing RPC migration such as polarization, directionality and chemosensitivity to steady state concentration gradients [50].

### Bridged µ-lane Chamber Fabrication

The framework of the bridged μ-lane device consists of two layers of poly-dimethylsiloxane (PDMS): a closed microchannel (95-μm-hydraulic diameter: 90μm-depth, 100μm-width; 1.3cm-length), a source reservoir (SRR) and a sink reservoir (SKR) (9μl volume each) on the bottom layer, and a source chamber (SRC) and sink chamber (SKC) (170μl volume each) connected by an open, hemispherical bridge channel (2-mm-depth; 9-mm-length). The system is fabricated using elastomeric molding of the silicon polymer PDMS (polydimethylsiloxane) and bonding of PDMS to PDMS and PDMS to glass. The SRC and SKC in the top layer are vertically and fluidically connected to the SRR and SKR in the bottom layer, and the bridge channel connects the SRC to the SKC in order to balance their solution volumes. The complete bridged μ-lane system is thus composed of the upper user interface layer with an open bridge channel that connects the SRC and SKC chambers, as well as a bottom layer closed microchannel that connects the SRR and SKR reservoirs [32]. The double-layered PDMS is then bonded to chemically cleaned (Nanostrip, Freemont, CA) glass slides using ozone gas exposure. 

### Operation of the Bridged µ-lane System

The bridged μ-lane system works by using the large volume chambers and bridge channel of the upper user interface layer to generate concentration gradients within the smaller volume microchannel on the bottom layer. After incubating cells along the microchannel, the cell culture media is manually loaded until it has filled the SRR, microchannel, SKR, SKC, and bridge channel. The test chemical solution is then loaded drop-wise into the SRC until the sample makes contact with the solution within the bridge channel to initiate system operation. The engineering design of the microchannel allows reagent perturbations of different concentrations of the test ligand to drive convective minuscule bulk velocity flow through the microchannel in the bottom layer, minimizing time required to attain a steady-state gradient of the ligand [28]. The volume of each chamber (170μl) is designed to be much larger than the volume within each reservoir (9μL) and μ-lane (0.1μl), in order to facilitate manual micropipette loading that initiates gradual transport into the microchannel with minimal channel entrance effects. Movement of chemicals from the source to sink reservoirs generates concentration gradients within the closed microchannel. Experimental measurement of EGF transport within this microchannel has previously been carried out and a diffusivity value of 2 X 10^-6^cm^2^/s was resolved for the ligand [28]. This value was then used to generate concentration profiles of EGF as a function of time and axial position, and it was determined that a steady state gradient of EGF was attained within this microchannel after 18hrs [28]. 

### Microfluidic Assay and Microscopic Imaging

The engineered microfluidic gradient assay adopts a previously established protocol [28]. The microchannel is coated with 10μg/mL laminin (Sigma-Aldrich Co., St. Louis, MO) for 1hr at room temperature. After aspirating excess unbound laminin, 0.1μl of RPC suspension (1×10^6^ cells/mL) in NB media supplemented with 10% FBS is then injected into the microchannel for a 4hr incubation period. The SRC, SKC and bridged channel are also filled with media. NB media concentrations containing 0 (control), 20, 40, or 400ng/ml EGF is then added drop-wise into the SRC till the solution makes contact with media in the bridge channel. The device is then mounted on the motorized stage of an inverted microscope (Nikon TE2000) housed in a humidified incubator (Okolabs, NA, Italy). The temperature in the incubator is maintained at 37°C with 5% CO_2_/balanced air supply. Live cell images were obtained at hourly intervals over 24hrs in three to five independent experiments per EGF test concentration. Emitted fluorescence from GFP^+^-RPCs is detected via a cooled CCD camera (Cool SNAP EZ, Photometrics, Tucson, AZ) and collated with Nikon software (Nikon Instrument Element 2.30 with 6D module, Morrell Instrument Co. Inc., Melville, NY). 

### Microfluidic Data Analysis

Cell motility parameters of center of mass (COM) and maximum accumulated distance, for each cell tracked over 24hrs, were resolved via sequential use of Nd-to-Image6d, Manual Tracking, and Chemotaxis and Migration Tool 2.0 (ibidi, Verona MI) plug-ins, all running on an ImageJ platform. COM is a strong parameter for evaluating chemotaxis, and measures the spatial average displacement of all cell endpoints with positive or negative coordinates, depending on the direction of movement of a single cell or a population of cells. Cell tracking data was further revised to select only video recordings of RPC movement between 10 and 24hrs to account for periods of attained steady-state gradients of EGF along the microchannel. Mean COM and maximum distance accumulated by RPCs were compared between control and test groups using the Dunnett statistical test. The Dunnett test was employed as a conservative statistical measure, appropriate for determining significant differences between treatment group means and a control mean rather than a total grand mean, especially when sample sizes among test groups are similar [51,52]. 

## Results

### Interactions of MAPK and JAK/STAT signaling pathways are predicted to govern EGF-induced RPC motility

**Figure 1:**
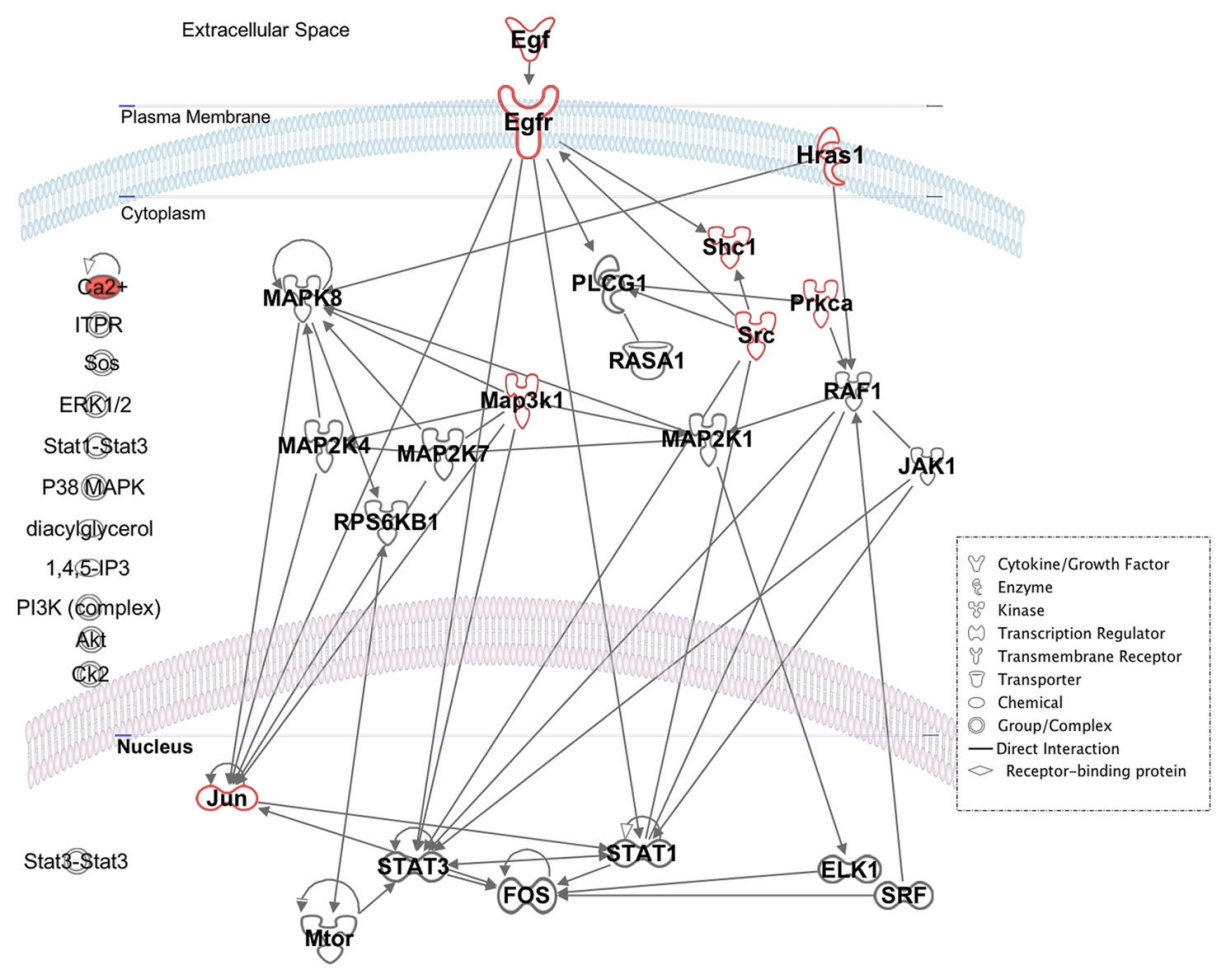
EGF Signaling Pathways driving migration of RPCs. Predicted network of direct molecular interactions influencing RPC migration in the mammalian retina. The network was generated using Ingenuity Pathway Analysis (IPA) bioinformatics tool. Molecular components of the EGF signaling pathway are localized to extracellular space, plasma membrane, cytoplasm and nucleus. Arrows indicate direct interactions between upstream and downstream pathway molecules. Molecule shapes outlined or filled in red denote significant association with chemotaxis as determined by a right-tailed Fischer test. Molecules displayed on the left represent key chemical groups predicted to participate indirectly in the network.

We used bioinformatics analysis to determine the presence of cell surface receptor and intracellular signaling pathways capable of driving RPC migration. Figure 1 displays a network model of direct molecular interactions predicted to be induced by EGF in mammalian neural retina. Network prediction was carried out using the pathway designer function of the Ingenuity Pathway Analysis (IPA) software and molecules relevant to chemotaxis function were identified by a right-tailed Fisher test for statistical significance (p=8.67E-10; molecules outlined or shaded in red). The right-tailed Fisher exact test is appropriate for bioinformatics data analysis and was used here to calculate *P* value to determine if associations between IPA resolved EGF network molecules and genes involved in chemotaxis were statistically significant [42,53]. Additional cellular functions resolved in this analysis included proliferation, cell-to-cell signaling, gene regulation and cell cycle progression. Our bioinformatics-derived network model also predicted two signaling pathways previously shown to be involved in EGF-induced motility (Figure 1). Activation of phospholipase C-γ1 (PLCG1) and subsequent hydrolysis of phospho-inositide bisphosphate (PIP) to yield products that activate protein kinase C (PRKCA) have been shown to increase cytoplasmic calcium levels which in turn stimulates cytoskeleton reorganization for cell motility [34,54,55]. PRKCA phosphorylates Raf1 (MAP kinase kinase kinase (MAP3K)) activating MAPK1/3 signaling [56]. Figure 1 also shows the coupling of EGFR activation to docking proteins GRB2 (Growth factor receptor bound)/SOS (Son of sevenless) via their SHC (Src homology) domain, an interaction that can activate HRas and its effector, the protein kinase Raf1. Raf can initiate phosphorylation events that activate the ERK1/2 signaling pathway leading to disruption of focal adhesions in mouse fibroblasts, and enhanced membrane ruffling, both essential processes for cell motility [34,55,57]. The same mechanism can phosphorylate insulin receptor substrate (IRS) and p85, which result in the activation of phosphoinositide 3-kinase (PI3K) [46], identified to be indirectly involved in the model network (Figure 1). Additionally, RAF1 directly associates with Janus Kinase (JAK) to activate Signal Transducer and Activator of transcription (STAT) proteins which can bind specific nuclear regulatory sequences to activate or repress transcription of target genes that are involved in cell motility [56]. 

Our bioinformatics model predicts a potential cross-interaction between the JAK/STAT and the EGFR tyrosine kinase (RTK)/Ras/MAPK pathways (Figure 1). Activated EGFR has previously been shown to promote JAK-independent tyrosine phosphorylation of STATs, a process possibly involving Src kinase [56]. MAPK has also been shown to specifically phosphorylate a serine residue near the C-terminus of most STATs as our model predicts. Although this phosphorylation event dramatically enhances transcriptional activity of STAT, it is not absolutely required for STAT activity [56]. Our bioinformatics model suggests that RPC motility signaling is a tightly regulated event involving a chemotactic role for EGF and the MAPK and JAK-STAT signaling pathways, with indirect involvement of the PI3K pathway. 

### EGF receptor is expressed and localizes to punctate structures along cellular processes in RPCs exposed to EGF

**Figure 2:**
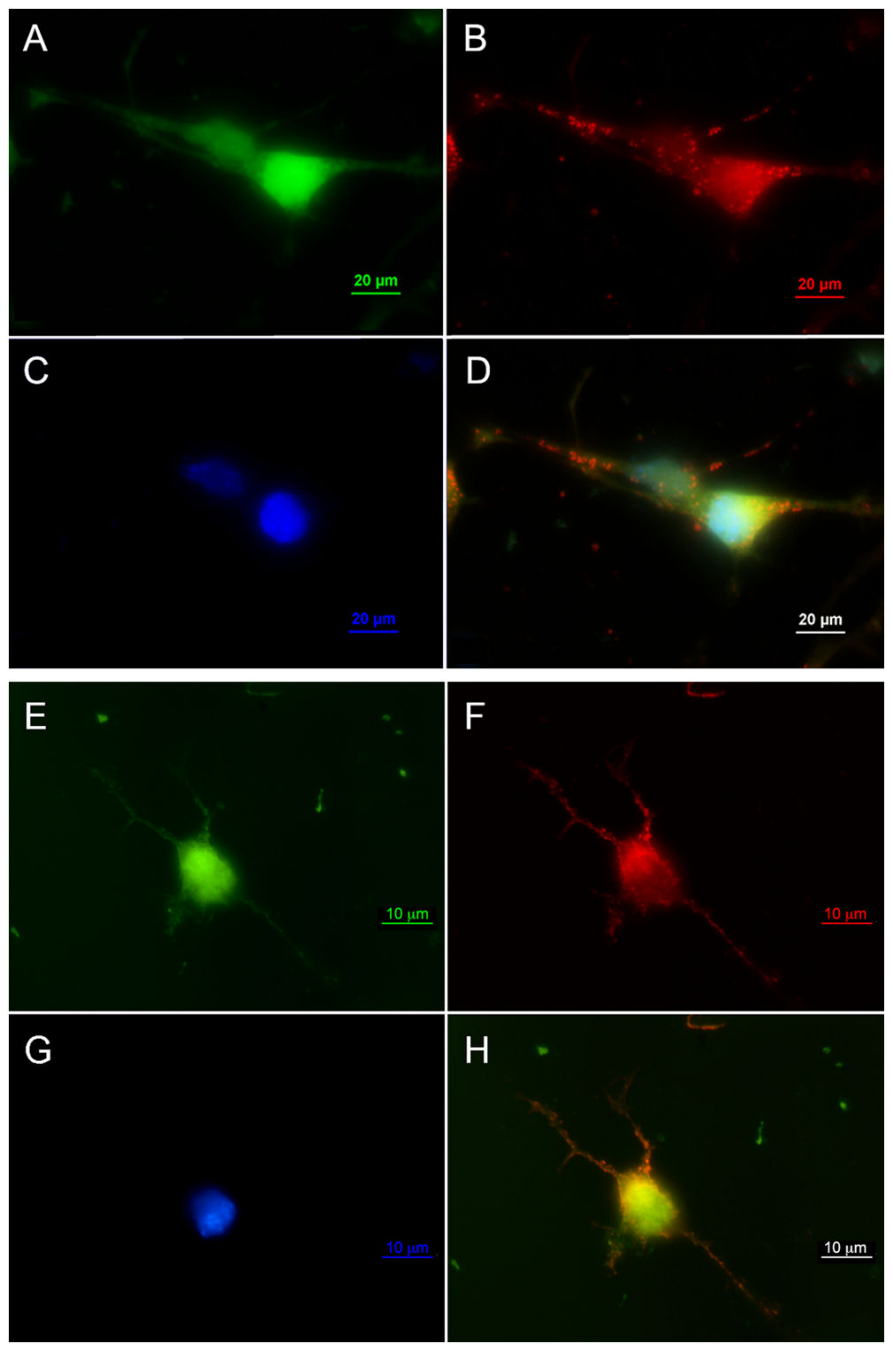
Immunocytochemical localization of RPC total and phosphorylated EGFR expression. Analysis of EGFR localization was performed on RPCs isolated from P3-5 transgenic mice expressing green fluorescent protein (GFP) on the actin promoter (actin-GFP). A) Actin-GFP RPCs express GFP ubiquitously revealing cytoplasm shape of two RPCs in 20ng/ml EGF, B) Rhodamine bound anti-total EGFR antibody staining reveals robust receptor localization to cell membrane of soma and processes. C) DAPI labeling of nuclei, D) Overlay of A-C. Panels E-F show identical imaging parameters as A-D, with the exception that F) utilizes anti-phosphorylated (activated) EGFR labeling. Scale: 10 microns.

To determine the expression and localization of EGFR in RPCs exposed to EGF, we performed immunofluorescence analysis of RPCs exposed to EGF. Antibodies recognizing both total EGFR and a phospho-specific form of EGFR were used (Figure 2B and 2F, respectively). Our results show that both non-phosphorylated and phosphorylated forms of EGFR are present in these RPCs and that the receptors localize throughout the cell body and also to punctate structures along cellular processes (Figure 2B,F). EGFR expression appeared identical between 20, 40 and 400 ng/ml EGF concentrations. No observable difference was detectable between experimental conditions for RPCs adherent to coverslips labeled with anti-total-EGFR and anti-phospho-EGFR. The labeling of EGFR observed in this work correlates with other studies showing similar results across a range of high and low EGF concentrations and in both RPCs and adult retinal cell types [17,40,58]. For this study, activated EGFRs represent the requisite cell surface mechanism to activate downstream JAK-STAT and PI3Kinase pathways involved in RPC motility.

### Motility tests support predicted role of JAK-STAT and PI3Kinase signaling in EGF-induced RPC migration

**Figure 3:**
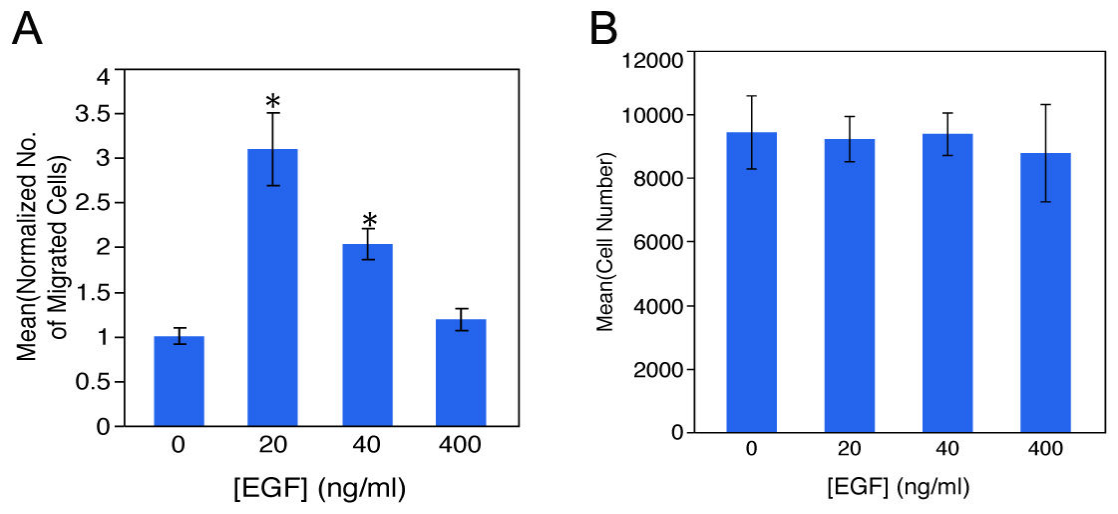
Boyden Chamber analysis of RPCs to establish optimal physiologic range of EGF concentrations. The Boyden chamber provided a high throughput screen to analyze EGF concentrations and exposure times. A) Demonstrates an optimal physiologic level of EGF, which facilitated significant RPC migration over 24hrs. Analysis of RPCs incubated in Boyden Chambers containing either 20ng/ml or 40ng/ml EGF revealed significant migration compared to control, 3.0981 ± 0.40 (p=<.0.0001*), 2.0340 ± 0.18 (p=0.0001*) respectively. While 400ng increased migration, this did not result in significant migration above control, 1.1885 ± 0.12 (p=0.7816). Pooled data from n=6 chambers are presented as normalized mean ± SEM. B) Analysis of RPC number following incubation in each EGF concentration revealed that there was no change in cell proliferation measured via MTT assay.

RPC motility in response to EGF concentrations ranging from 20ng to 400ng/ml was tested using a modified Boyden chamber assay. The 24hr duration of the experiment employed was optimal for ligand-induced motility of RPCs as determined by initial Boyden chamber assay optimization studies spanning 6 to 48hrs. The range of EGF concentrations tested in this study have previously been shown to drive motility in neuronal cell types in Boyden chamber experiments and are relevant to the retinal microenvironment [24,28,59,60]. Using Dunnett analysis, significant increases in RPC migration were observed in 20ng/ml and 40ng/ml experimental conditions compared to control (n=6 Boyden Chambers each). For each condition, analysis is reported as normalized mean number of migrated RPCs, SEM and p-value: 0ng/ml = 1.0000 ± 0.09 (p=1.0000), 20ng/ml =3.0981 ± 0.40 (p=<.0.0001*), 40ng/ml = 2.0340 ± 0.18 (p=0.0001*) and 400ng=1.1885 ± 0.12 (p=0.7816). Results depicted in Figure 3A show a significant increase in RPC motility in response to 20ng/ml (p<0.0001) and 40ng/ml (p=0.0001) of EGF compared to control over 24hrs. In contrast, there was no significant change in motility of RPCs exposed to 400ng/ml of EGF (Figure 3A). The Boyden chamber results were independent of RPC growth rates based on MTT proliferation analysis (Figure 3B). These results support the IPA interactions network model predictions of EGF-induced motility of RPCs. 

**Figure 4:**
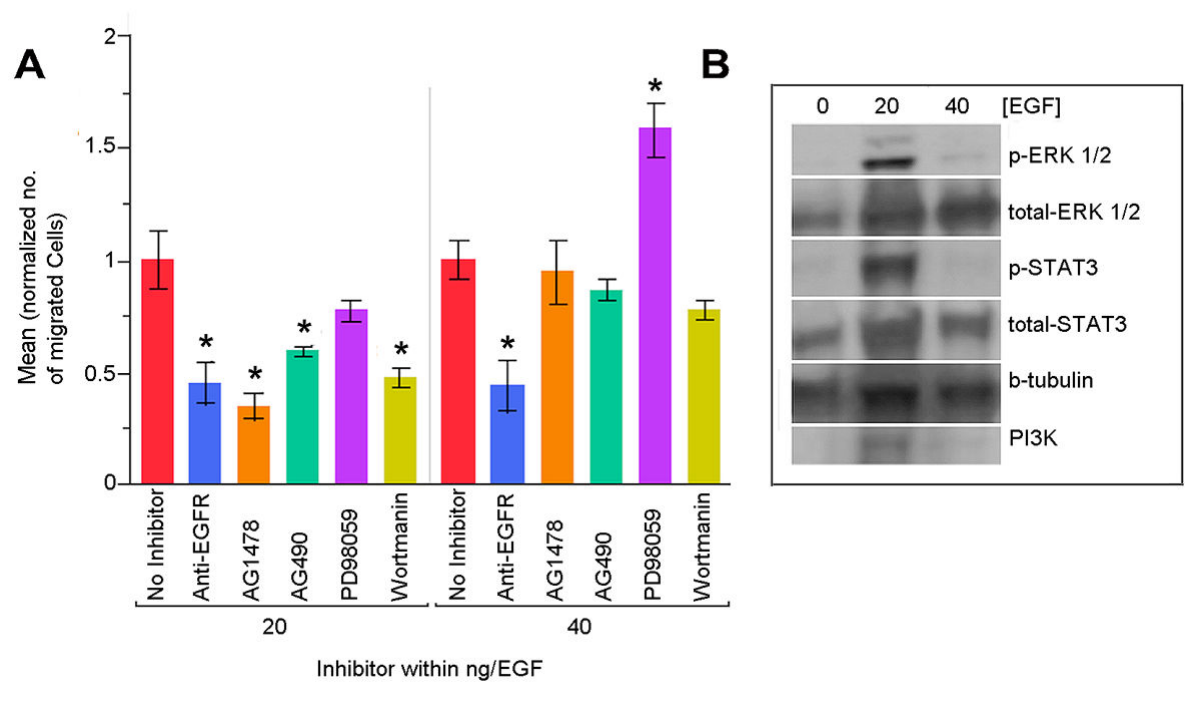
Pharmacologic inhibition of Signaling Pathways Predicted to influence RPC migration. RPCs were treated with selected inhibitors of signaling pathways identified in IPA bioinformatics analysis and evaluated for migration over 24hrs using n=6 Boyden Chamber assays per inhibitor. A) Analysis of RPC migration following pre-treatment with selected inhibitors reveal that significant inhibition of migration was observed in low 20ng EGF condition using anti-EGFR (0.4499 ± 0.10 SEM (p=<.0001*) to antagonize EGFR cell-surface binding, AG1478 0.3427 ± 0.06 SEM (<.0001*) to inhibit phosphorylation of the EGFR cytoplasmic domain, AG490 (0.5913 ± 0.02 SEM (p=0.0012*) to inhibit STAT3 activity and Wortmanin (0.4699 ± 0.04 SEM (p=<.0001*) to inhibit PI3K activity. The use of PD98059 to inhibit ERK1/2 activity did not result in significant inhibition of migration. In the presence of a higher 40ng/ml concentration of EGF, significant inhibition of migration was observed with anti-EGFR (0.4370 ± 0.12 SEM (p=0.0002*), while PD98059 1.5810 ± 0.12 SEM (p=0.0001*) increased migration. Statistical significance was determined using Dunetts analysis and data are presented as normalized mean. B) Robust activation of identified signaling pathways was observed in 20ng/ml EGF concentrations. Western Blot analysis demonstrated the presence of both non-phosphorylated (inactivated) and phosphorylated (activated) STAT3, ERK1/2 and P13K proteins RPCs. Inhibition with significant p-values are denoted with an asterisk.

To identify which molecules in the EGF signaling pathway contribute to RPC motility, inhibition studies were performed based on signaling pathways identified in initial bioinformatics analysis at 20ng and 40ng/ml concentrations shown to be significant in Boyden Chamber studies. RPCs were treated with inhibitors prior to the 24hr motility assay. We compared RPC migration from two independent experiments for 20ng/ml 40ng/ml EGF and controls (n=6 chambers each) with each pharmacologic inhibitor. Statistical significance was determined using the Dunnett test. For each condition, results are reported as normalized mean migrated RPC, SEM and p-value: In 20ng/ml condition, no Inhibition = 1.0000 ± 0.08 (p=1.0000), PD 98059 = 0.7722 ± 0.05 (p=0.1341), AG 490 = 0.5913 ± 0.02 (p=0.0012*), Wortmanin = 0.4699 ± 0.04 (p=<.0001*), Anti-EGFR = 0.4499 ± 0.10 (p=<.0001*) and AG 1478 = 0.3427 ± 0.06 (<.0001*). At the 20ng/ml level, the majority of inhibitors effectively reduced migration. RPCs in the 40ng/ml condition appeared to exhibit a decreased response to selected inhibitors: in 40ng/ml, no Inhibition = 1.0000 ± 0.09 (p=1.0000), PD 98059 = 1.5810 ± 0.12 (p=0.0001*), AG 490 = 0.8619 ± 0.04 (p=0.7727) Wortmanin = 0.7779 ± 0.04 (p=0.3368) Anti-EGFR = 0.4370 ± 0.12 (p=0.0002*) and AG 1478 = 0.9433 ± 0.14 (p=0.9933). To determine if ligand binding to EGFR and receptor activity influenced observed increases in RPC motility, we incubated cells with either a monoclonal antibody to EGFR (anti-EGFR) to inhibit EGF binding to its receptor or a specific EGFR tyrosine kinase inhibitor (Tyrphostin AG1478) to inhibit phosphorylation of the receptor cytoplasmic domain (Figure 4A). Our results show that inhibition of ligand binding to EGFR or receptor tyrosine kinase selectively inhibited motility of RPCs exposed to 20ng/ml EGF but not to 40ng/ml EGF (Figure 4A). To determine which downstream signaling pathways were involved in the EGF-induced motility, we also treated cells with inhibitors either to STAT3 (AG490), ERK1/2 (PD98059), or PI3K (Wortmanin). Results in Figure 4A show that inhibition of the JAK-STAT and PI3K pathways led to a significant reduction in RPC motility in response to 20ng/ml EGF. A significant change was not observed in cells treated with 40ng/ml of EGF. Inhibition of ERK1/2 of the MAPK signaling pathway did not significantly alter RPC motility in either 20 or 40ng/ml of EGF (Figure 4A). 

Using western blot analysis, the activation states of ERK1/2, STAT3, and PI3Kinase intracellular proteins in RPCs incubated in either 20 or 40ng/ml of EGF for 24hr were determined. Our results show that all three proteins were phosphorylated in RPCs incubated with 20ng/ml but not 40ng/ml of EGF (Figure 4B). Immunofluorescence results in Figure 2 confirm the presence of phosphorylated EGFR in RPCs exposed to 20ng/ml. These findings suggest that 20ng/ml EGF optimally stimulates EGFR tyrosine kinase activity and cell motility in our RPC population. The intracellular signaling cascades involving JAK-STAT and PI3K signaling appear necessary for EGF-induced RPC motility. The central role of ERK1/2-associated signaling predicted by our model network (Figure 1) may explain the activated state of the protein in RPCs incubated in 20ng/ml EGF.

### EGF gradients stimulate chemokinetic migration of individual RPCs in bridged μ-Lane microfluidic system analysis

**Figure 5:**
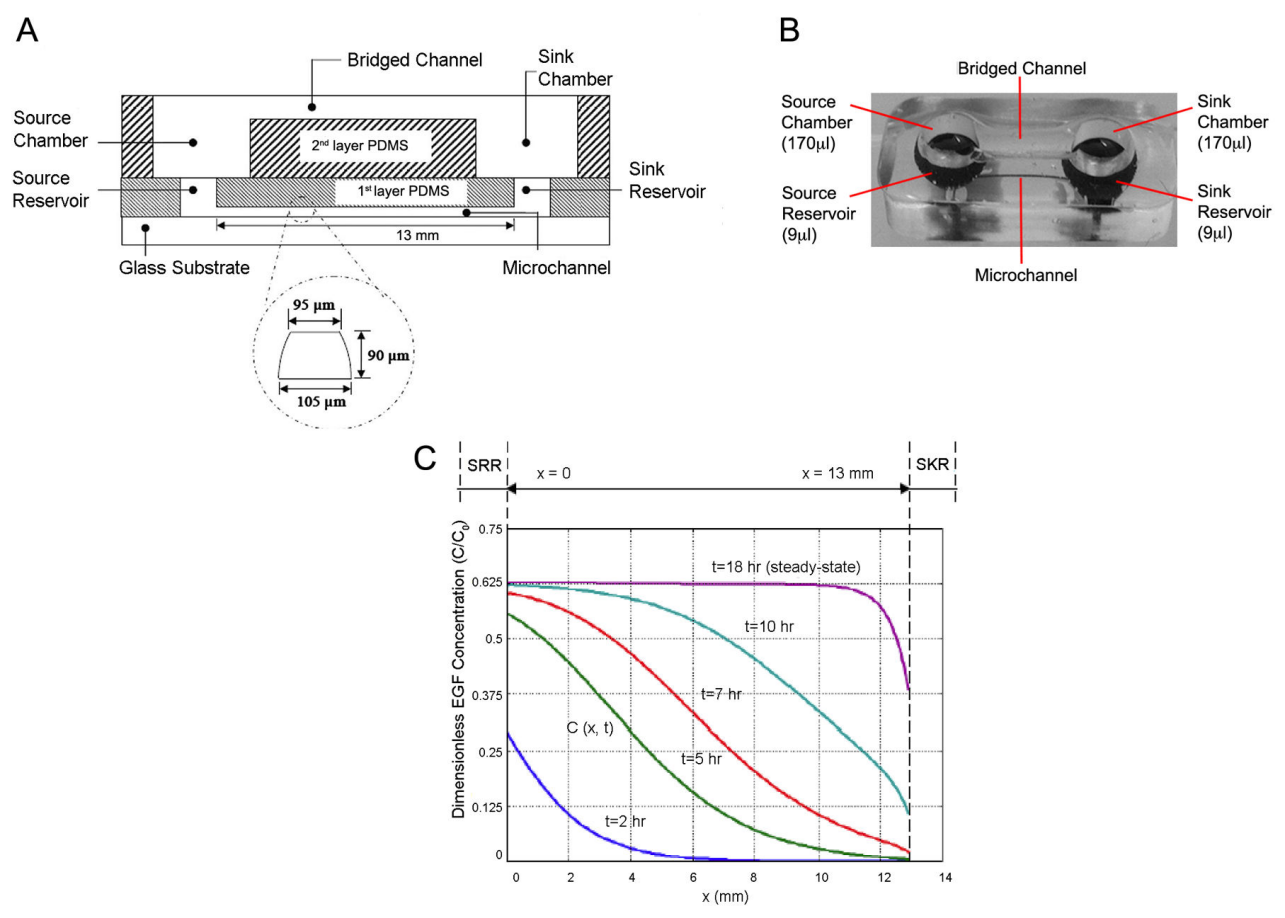
Microfluidic design and modeled gradients of bridged μ-Lane system. Microfluidic analysis provides precise control over the establishment and maintenance of chemical concentration gradients. Microfluidic function incorporates fluid dynamics, molecular diffusivity over time and hydrostatic fluid balance within all parts of the system. A) The microfluidics design includes a microchannel measuring approximately 13mm (length), 90μm (depth), and 100μm (width), with two reservoirs (source and sink) defined as the first layer PDMS. A second layer of PDMS, the user interface layer, consists of a bridge channel and two chambers (source and sink) bonded on top of the first layer forming a fluidic connection between both layers. B) Double-layer PDMS microfluidic device fabricated after soft lithography and bonding onto glass slides with volume capacities of the chambers and reservoirs indicated. C) Normalized EGF concentrations plotted against increasing distance from the source reservoir (SRR) to the sink reservoir (SKR) as a function of time.

**Figure 6:**
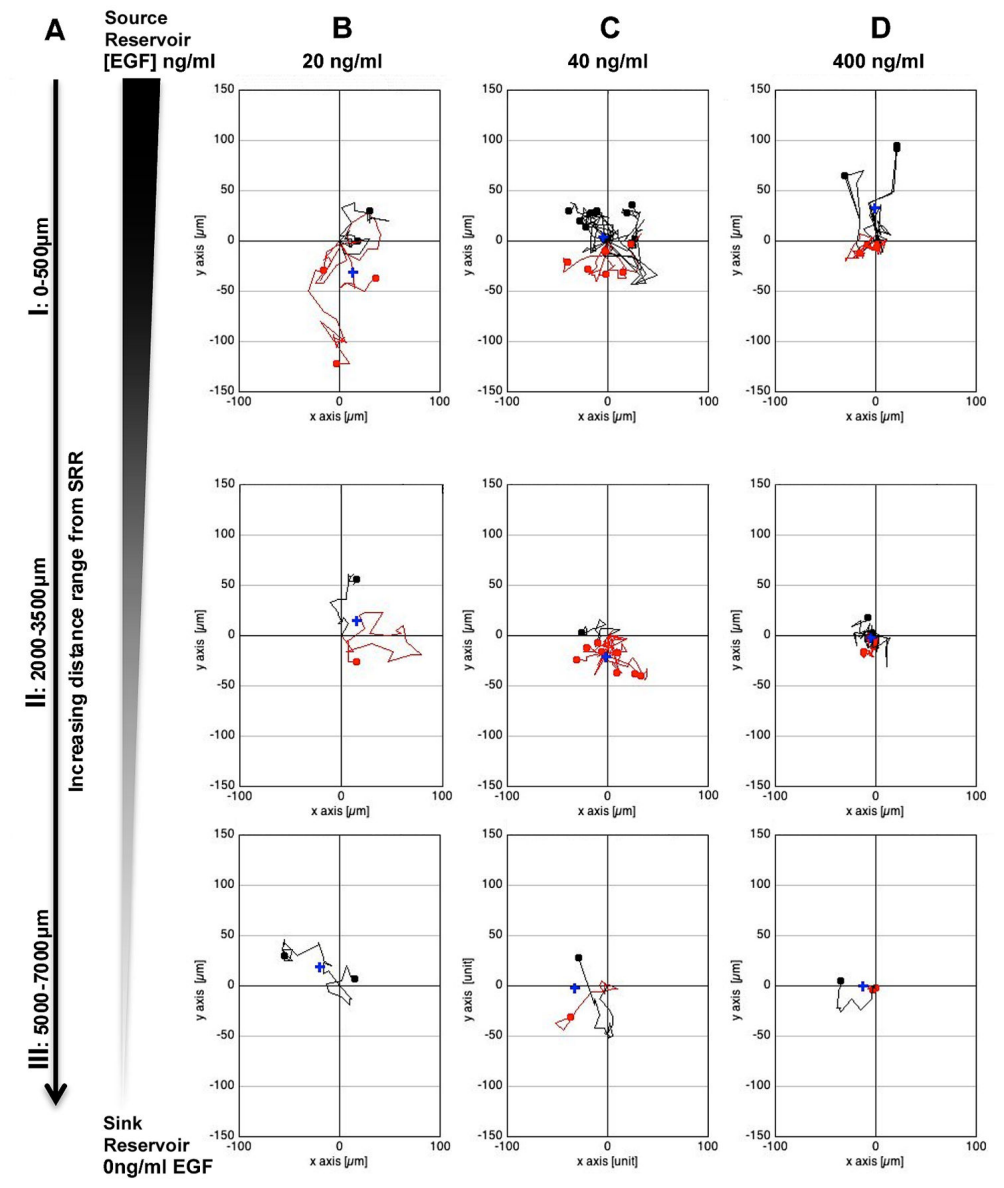
Trajectory analysis paradigm for RPC migration in microfluidic gradients at increasing distances from the EGF source. Positional tracking of RPCs migrating in steady state gradients of EGF generated in a 13mm bridged μ-lane device over a 24hr period was visualized using Ibidi Migration software to generate wind-rose plots (A-D). A) Cell tracking results are depicted at increasing distances from the source reservoir: 0-500μm (I), 2000-3500μm (II), 5000-7000μm (III). B-D) Depiction of RPC trajectory plot mapping in steady-state gradients of 20, 40, and 400ng/ml EGF analyzed from source reservoirs to sink reservoirs. A trend of reduced chemokinetic migratory dynamics can be observed with increasing EGF concentrations up to 400ng/ml. Red and black traces indicate RPCs with negative and positive COM, respectively.Statistics resulting from trajectory data depicted here analyzed for maximum distance and center of mass are reported below. The x- and y-axis denote RPC displacement in horizontal and vertical directions, respectively.

**Figure 7:**
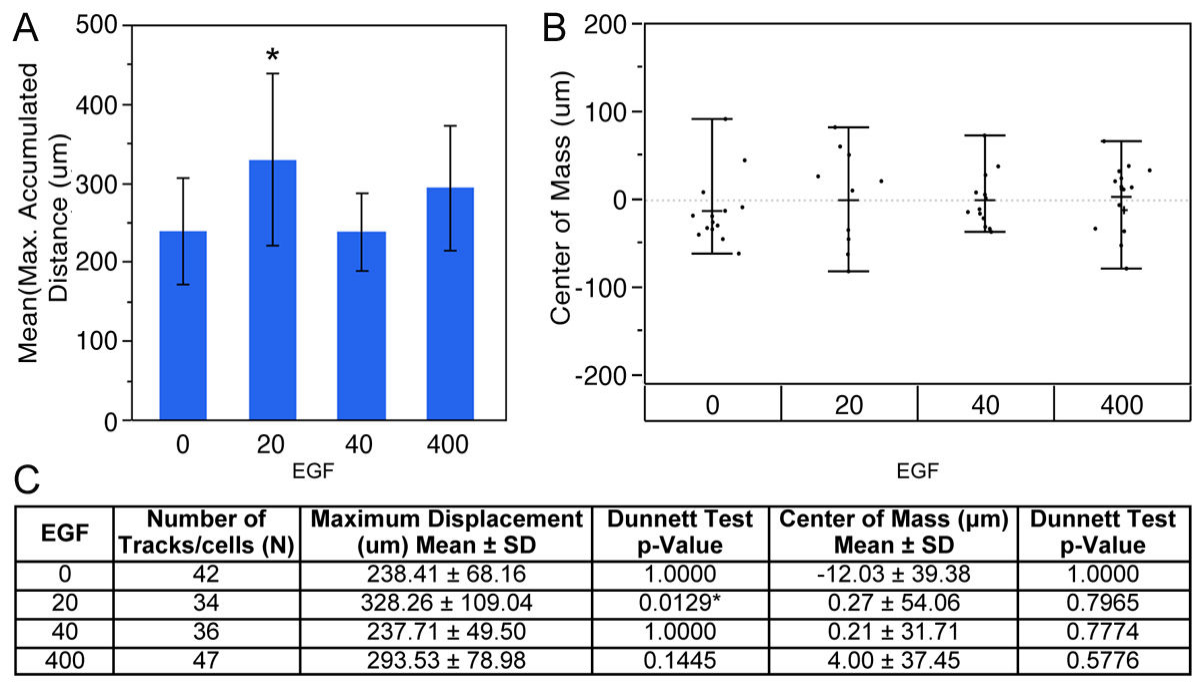
Analysis of RPC maximum accumulated distance and center of mass dynamics in microfluidic bridged μ-lane EGF gradients. A) RPC maximum displacement was analyzed in the absence or presence of varying EGF gradients measured post-steady-state within a 24hr period, and results are expressed as mean ± standard deviation for three independent studies per EGF concentration. Only RPCs cultured in 20ng/ml EGF exhibited significantly higher maximum displacement compared to cells in the control microchannel (328.26 ± 109.04, p=0.0166), Dunnett Test. B) Similar measurements for center of mass (COM) of RPCs yielded non-significant differences in both means and medians among test groups (Brown-Forsythe, p=0.1622) indicating no significant chemotactic or directed migration between EGF concentrations. This reveals that the increased Maximum accumulated distance response can be defined as chemokinetic. C) displays averaged maximum distance accumulated and COM of RPCs in defined EGF concentration gradients with their respective standard deviation and p-values results from the Dunnett statistical test. Significant p-values are denoted with an asterisk.

The Boyden chamber assay is best suited for studying ligand-induced mass movement of cells, as detailed individual cell migratory dynamics cannot be measured. The Boyden chamber is comprised of an upper well separated from a lower well by a micropore filter. Cells are added to the upper well and chemotactic molecules to the bottom well. Following incubation, the total number of cells migrated through the micropores toward the lower well are quantified [47-49,61]. Temporal control and quantitation of stable gradients are difficult to accomplish in Boyden chamber assays [62]. In contrast, we are able to show that in a 13mm long, bridged μ-lane system the concentration of EGF varies in a mathematically defined manner, is spatially and temporally quantifiable, and becomes constant for a defined period, once steady-state gradients are achieved (Figure 5C and [32]). A number of studies have shown that EGF is capable of stimulating motility in the form of chemotaxis (directional migration) and chemokinesis (random migration) [23,24,63]. The employment of the bridged μ-lane system has enabled time-lapse imaging of individual RPC responses to different gradients and concentrations of EGF along the microchannel, providing data to distinguish between chemotactic and chemokinetic migratory dynamics (Figure 6). After exposing RPCs to gradients of 20, 40 and 400ng/ml EGF in the microchannel system, we analyzed accumulated maximum distance of migration and single cell center of mass (COM) directionality. COM defines the average point of all cell endpoints and quantifies migration direction. Analysis of three independent microfluidic experiments per condition revealed that RPCs exposed to gradients generated from 20ng/ml EGF exhibited significantly higher motility including accumulated maximum micron distances (328.3μm ±109.04 SD) compared to control channels without EGF (238.4μm ±68.16 SD) (Dunnett test, p=0.0129) (Figure 7). Accumulated distance migrated by RPCs in 40ng/ml EGF (237.71μm ± 49.50 SD) and 400ng/ml EGF (293.53μm ± 78.98 SD) microchannel conditions compared to control did not yield significant differences, p= 1.0000 and p= 0.1445 respectively. Analysis of center of mass (COM) of RPCs yielded non-significant differences in both medians (Brown-Forsythe, p=0.1622) and means among test groups indicating no significant chemotactic or directed migration between EGF concentrations compared to control: 20ng/ml 0.27 ± 54.06 p=0.7965, 40ng/ml 0.21 ± 31.71 p=0.7774 and 400ng/ml 4.00 ± 37.45 p=0.5776 (Figures 6,7). In Figure 6, chemokinetic motility is visualized in trajectory plots of RPC movement tracked for 24hrs across microfluidic EGF gradients. In Figure 6, red trajectories depict negative COM or cell movement away from the EGF source reservoir while black trajectories depict positive COM or cell movement towards the source reservoir. Greater multi-directional displacement of RPCs in the 20ng/ml EGF condition can be observed in this figure compared to other EGF gradient conditions. The degree of chemokinetic movement is also seen to decrease with increasing distance from the EGF source reservoir. A sample time-lapse video recording corresponding to the trajectory plot of RPC movement in EGF-gradients (Figure 6) is shown in movie S1.

## Discussion

### Bioinformatics Modeling of Signaling Networks

To refine targets from the wide range of biochemical signaling molecules involved in neural progenitor migration, we performed bioinformatics analysis using IPA software. Our bioinformatics-derived network molecules identified two signaling pathways previously shown to be involved in EGF-induced motility (Figure 1). Gene network databases have been used to predict connectivity of genes involved in chemotactic pathways of migrating adult neural progenitors to specific lamina [64,65]. Bioinformatics approaches have also been used to validate molecular network interactions directing adult neuronal progenitor chemotaxis during neurogenesis in the sub-ventricular zone and cortex [39,66-68]. In this study, the bioinformatics predicted EGF signaling network resulted in valid molecular targets that were shown to influence RPC motility at the receptor and second-messenger level in Boyden chamber and microfluidic devices.

### Boyden and Microfluidic Approach

Our Boyden analysis demonstrated that EGF stimulates migration of RPCs at selective uniform concentrations (Figure 3). In Boyden chamber assays, steep gradients of EGF generated at the start of experiments dissipate and rapidly become uniform. We utilized Boyden analysis as a high-throughput screen of multiple concentrations and incubation times. In contrast, microfluidic analysis provides steady-state gradients maintained along the length of the microchannel as a function of time. The bridged µlane system can sustain steady state concentrations and concentration gradients of ligands over time spans of 48-96 hours. The double-layered PDMS design exploits the ultralow bulk velocities generated by density differences of test ligand concentrations that ensure continuous one-dimensional transport within the microchannel by convective diffusion. Numerical simulation of mass transport within the bridged µlane system combines quantification of 2D continuity, convective-diffusion, momentum, and hydrostatic pressure that are computed using the Finite Element Method [32]. Previously, the microfluidic device has been used to generate gradient profiles that span over five orders of magnitude and over time scales that approach the microenvironments generated *in vivo*. Microfluidic technology has proven highly valuable in the creation of biomimetic microenvironments [32] and has been used to assess proliferative, survival and differentiation responses of neural progenitor cells to concentration gradients of extracellular signaling molecules [27,69]. In this study, live-cell time-lapse microchannel data was analyzed for maximal accumulated distance and COM. The data revealed that steady-state EGF gradients were capable of influencing RPC motility and that chemokinesis was enhanced at low EGF concentrations (Figure 6, 7).

### RPC Motility is influenced by low EGF ligand concentrations

Earlier investigations using retinal Muller glia and neurons revealed that binding of EGF to its receptor is highly specific, concentration-dependent, and saturates at approximately 80-100ng/ml EGF [19,70,71]. Specificity of EGF binding has been associated with higher numbers [70] and/or varying states of EGFRs [40,71]. High and low affinity receptor states differ in their associations with the EGF ligand, and their activation differentially influences cellular function [40,70,71]. The number of high-affinity receptors has been suggested to be 5-10% of the total EGFR number [40,58,71]. Similarly, the type and proportion of EGFRs recruited and activated may differ with varying concentrations of EGF. Low levels of EGF have been reported to bind primarily high affinity receptors which trigger intracellular signaling proteins including Erk, Akt, Shc1, CrkL and Gab1, while higher concentrations of EGF bind low-affinity receptors activating PLCγ1 and the Stat proteins [40]. Low- and high-affinity EGF receptors are derived from the same mRNA transcript and low affinity receptors can be converted to high affinity receptors to modify cell response dynamics [40]. Low-affinity receptor activation has been shown to modify integrin receptor levels and decrease cell adhesion. EGF concentrations capable of activating both high and low-affinity EGF receptors have been shown to facilitate cell motility in vitro *and in vivo* [22,23,40]. Our results showing stimulation of RPC migration at low EGF concentrations of 20ng/ml and 40ng/ml suggest that the increased motility is mediated in part by high-affinity receptor signaling. Further analysis could reveal specific ratios of high and low affinity EGFR dynamics involved in RPC motility.

### RPC Motility is Directed by the EGFR JAK-STAT Pathway

Our results showing reduced motility of RPCs in response to steric inhibition of EGFR and chemical inhibition of the receptor activity strongly suggest the involvement of EGFR and downstream activity in EGF-induced RPC motility. EGF signal transduction pathways are shaped by interactions of many components of signaling networks. A subtle difference in input signals and/or interaction kinetics may result in differential response patterns. The kinetics (i.e. the transient and steady-state behavior) of the cellular response to EGF depends on many factors, including the number of receptors displayed on the cell surface; the concentration of the growth factor, docking, and target proteins; and their initial activity states [72]. Moreover, other signaling pathways that share or interact with one or more components of the EGFR pathway can influence the kinetic pattern of EGFR signaling [73]. EGF receptors form either homo- or hetero-dimers following ligand binding, each dimer showing different affinity for ligands and different signaling properties. Often, components of different pathways interact, resulting in complex signaling networks, components of which are described in Figures 1 and 4. These networks exhibit emergent properties such as integration of signals across multiple time scales, generation of distinct outputs depending on input strength and duration, and self-sustaining feedback loops. Furthermore, there are slower processes involving receptor internalization and its subsequent degradation in lysosomes, which have an important role in EGF-induced signaling [72,74]. In this study, RPCs stimulated with 20ng/ml of EGF expressed activated forms of EGFR, ERK1/2, STAT3 and PI3K. Analysis of Inhibition of individual downstream signaling pathways suggests that EGF-stimulated motility involves the JAK-STAT and PI3K pathways and not the ERK1/2 pathway. These findings suggest differential activation and inhibition of canonical EGF signaling pathways. Additional studies could explore additional factors involved in EGF signaling including multiple interacting ligands; receptor density/degradation and overlapping signaling network dynamics. 

### RPCs Show Chemokinetic Migratory Response in low nanomole EGF Microfluidic Gradients

To study the mode of individual RPC migration in response to defined EGF gradients we utilized engineered microfluidic devices. Defined steady-state gradients generated using microfluidics provide mathematically modeled, quantitative information about ligand concentrations in a controlled environment. By carefully choosing the concentration of the input, a wide variety of gradient steps and concentration ranges can be created in microfluidic devices [62]. Applying a 20ng/ml EGF media concentration in the bridged μ-lane device stimulated the greatest chemokinetic responses at regions closest to the source reservoir (SRR), where the chemical gradient is steepest (Figure 5). The ligand concentration at 18hrs in the region of the channel closest to the SRR is approximately 63% of the starting EGF concentration [28] suggesting that RPCs respond to steep gradients of low EGF concentrations. The results are supported by several studies describing saturating effects of EGF in signal transduction and motility [28,40,70]. Our study is the first demonstration of the effect of controlled gradients on RPC motility *in vitro*. 

### Future Work

Findings from this study defining EGF receptor ligand interactions and emergent motility dynamics may be useful towards developing *in vitro* models of endogenous signaling guiding transplanted RPC motility *in vivo*. Sub-retinally transplanted RPCs migrate through the interphotoreceptor matrix to reach appropriate retinal lamina for repair. To accurately model RPC migration, future microfluidics studies may replicate the topological and biochemical properties of the interphotoreceptor matrix by employing three-dimensional biomimetic extracellular matrix gel composed of laminin, fibronectin and collagen proteins as well as chondroitin sulfate proteoglycans and glycosaminoglycans [75,76]. An ECM substrate available for initial testing is MaxGel, and engineered hydrogels can be covalently modified with chondroitin and heparin-sulfate proteoglycans and peptides [77,78]. Transparent synthetic ECM matrices form porous soft gels along the length of the microchannel, allowing formation of a chemical gradients and visual tracking for quantitative assessment of cell morphology and motility. A number of recent studies have shown that stem and progenitor cells cultured in 3D gels attach to and remodel their extracellular matrix environments allowing for proliferative and migratory dynamics comparable to those observed in vivo [79-81]. The use of 3D gels to mimic the retinal microenvironment could enhance future in vitro models analyzing RPC motility.

In summary, we present experimental work showing a mode of migration of RPCs in microfluidic environments, which was previously not described. We demonstrate that EGFR activation of downstream JAK-STAT signaling pathways in our population of RPCs is optimal at low EGF concentrations. The inhibition of these pathways results in inhibition of migration. In addition live-cell imaging of RPCs in defined chemical gradients demonstrates that EGF induces chemokinetic migratory dynamics. This work represents an important step in the analysis of transplantable RPC populations in physiologic concentrations comparable to those found in developing and disease retina [17]. The data presented here provides a model of stem and progenitor cell migratory analysis to inform future transplantation strategies. 

## Supporting Information

Movie S1 **Time-lapse microscopy of RPC migratory response to EGF in a microfluidic channel.** Live-cell images were obtained at hourly intervals over 24hrs. GFP^+^-RPC fluorescence was detected using a CCD camera coupled to a Nikon Ti microscope with Elements software. Visualized RPC migratory trajectories were then quantified for accumulated distance and center of mass motility.
(ZIP)
