# Correction: Microfluidic Generated EGF-Gradients Induce Chemokinesis of Transplantable Retinal Progenitor Cells via the JAK/STAT and PI3Kinase Signaling Pathways

**DOI:** 10.1371/annotation/ec7d8834-50c9-4e2c-b22e-5b73e8b1377c

**Published:** 2014-01-17

**Authors:** Uchenna J. Unachukwu, Moira Sauane, Maribel Vazquez, Stephen Redenti

A funding organization and grant for the third author (MV) are incorrectly omitted from the Funding statement. The Funding statement should read: 

"This work was supported by a National Institute of General Medical Sciences (NIGMS) GM096935 grant (SR) and National Science Foundation (NSF) CBET 0939511(MV). The funders had no role in study design, data collection and analysis, decision to publish, or preparation of the manuscript."

